# Regulation of the X Chromosome in the Germline and Soma of *Drosophila melanogaster* Males

**DOI:** 10.3390/genes9050242

**Published:** 2018-05-04

**Authors:** Eliza Argyridou, John Parsch

**Affiliations:** Division of Evolutionary Biology, Faculty of Biology, LMU Munich, Grosshaderner Str. 2, 82152 Planegg-Martinsried, Germany

**Keywords:** X chromosome, X suppression, germline, soma, dosage compensation, *Drosophila melanogaster*

## Abstract

During the evolution of heteromorphic sex chromosomes, the sex-specific Y chromosome degenerates, while the X chromosome evolves new mechanisms of regulation. Using bioinformatic and experimental approaches, we investigate the expression of the X chromosome in *Drosophila melanogaster*. We observe nearly complete X chromosome dosage compensation in male somatic tissues, but not in testis. The X chromosome contains disproportionately fewer genes with high expression in testis than the autosomes, even after accounting for the lack of dosage compensation, which suggests that another mechanism suppresses their expression in the male germline. This is consistent with studies of reporter genes and transposed genes, which find that the same gene has higher expression when autosomal than when X-linked. Using a new reporter gene that is expressed in both testis and somatic tissues, we find that the suppression of X-linked gene expression is limited to genes with high expression in testis and that the extent of the suppression is positively correlated with expression level.

## 1. Introduction

In many animal species with a long evolutionary history of having separate sexes, heteromorphic sex chromosomes are thought to have evolved when an ancestral pair of autosomes gained a sex-determining locus [1,2]. During this early stage of sex chromosome evolution, the accumulation of sexually antagonistic mutations (mutations that are beneficial to one sex but detrimental to the other) in the region surrounding the sex-determining locus provides selective pressure to inhibit recombination between the homologous chromosomes, as it would be deleterious for such mutations to be expressed in the wrong sex. This process can eventually result in a sex-specific chromosome that does not recombine over most, or even all, of its length [3,4]. The absence of recombination allows for the accumulation of deleterious mutations and repetitive sequences, leading to gene loss and heterochromatinization [5,6]. In taxa with very old sex chromosomes, the degeneration of the chromosome lacking recombination may be nearly complete. For example, in *Drosophila melanogaster*, only 12 protein-coding genes have been identified on the Y chromosome, while over 2100 protein-coding genes are present on the X chromosome [7]. The *D. melanogaster* Y chromosome does not share any homologous genes with the X chromosome, suggesting that it has lost all of its ancestral gene content and gained new genes that are derived from autosomal sequences [8,9,10,11]. 

Degeneration of the Y chromosome causes X-linked genes to be present in only one copy in males, leading to an imbalance with autosomal genes that are present in two copies. To compensate for this difference in gene dose, special regulatory mechanisms have evolved. In the soma of *D. melanogaster*, an RNA-protein complex known as the dosage compensation complex (DCC) binds specifically to the male X chromosome and increases the expression of X-linked genes roughly two-fold [12,13,14]. This balances the average expression of X-linked and autosomal genes in males and of X-linked genes between males and females. A comparison of global gene expression in the testis, ovary, and thorax suggested that X chromosome dosage compensation is absent in the male germline [15,16], although there is some debate regarding this issue [17,18]. Studies comparing the expression of reporter genes and transposed genes in testis have found that the expression of X-linked genes is significantly lower than that of autosomal genes, even after accounting for gene dose [15,19,20,21,22]. This suggests that there is a mechanism to suppress expression from the X chromosome in the male germline that is analogous to the meiotic sex chromosome inactivation (MSCI) that occurs in mammals [23,24]. A recent study reported that there is a positive correlation between the degree to which an X-linked gene is suppressed in the male germline and its expression level when relocated to an autosome [22], although this was based on expression data from a relatively small number of transposed genes and has not been confirmed by experimental studies (e.g., reporter genes).

The present study further investigates gene regulation of the *D. melanogaster* X chromosome. We use recently published expression data [25] from male and female gonads and different somatic tissues/body segments (head, brain, midgut, hindgut, and Malpighian tubule) to compare the expression of X-linked and autosomal genes. We find evidence for nearly complete X chromosome dosage compensation in somatic tissues, although genes with very high expression are underrepresented on the X chromosome. Dosage compensation appears to be absent in testis and there is a much greater underrepresentation of X-linked genes with high expression in this tissue, suggesting that there may be an upper limit to X-chromosomal expression. Experimental results from a new reporter gene with relatively low expression in testis are consistent with this interpretation: in contrast to reporter genes with high expression in testis, it does not show reduced expression when X-linked. Our results offer a new insight into the regulation of X-linked genes in the male germline by providing evidence for the interplay between expression level and X suppression.

## 2. Materials and Methods

### 2.1. High-Throughput Expression Data

RNA-sequencing (RNA-seq) expression data for *D. melanogaster* testis, ovary, and five somatic tissues/body segments of males and females (head, brain, midgut, hindgut, and Malpighian tubule) were obtained from FlyAtlas2 [25]. The data were downloaded and extracted from a MySql database (version FlyAtlas2_17.10.07.sql). The expression level of each gene in each sex was calculated as the mean of the three biological replicates performed for each tissue in units of fragments per kilobase of transcript per million mapped reads (FPKM). Only genes with FPKM > 1 were included in our analysis. Within each sex and tissue, differences in expression between autosomal and X-linked genes were tested using a Wilcoxon test implemented in R [26]. Under- or over-representation of X-linked genes within different expression categories was tested with a χ^2^ goodness-of-fit test implemented in R [26]. For this, the expected proportion of X-linked genes in each category was determined from the ratio of X-linked to autosomal genes in the entire FlyAtlas2 dataset.

### 2.2. Reporter Gene Mobilization

The reporter gene construct we used is part of a transposable element vector that was described previously [27]. In this construct, terminal sequences of a *P*-element flank two copies of the reporter gene and the *D. melanogaster mini-white* eye color marker gene. Each copy of the reporter gene consists of a human cytomegalovirus (CMV) promoter directly proximal to the *Escherichia coli lacZ* coding sequence. The CMV promoter is capable of driving expression of the reporter gene in multiple *D*. *melanogaster* tissues [27]. Tandem copies of the reporter gene were used in order to ensure sufficient expression within individual tissues (e.g., testis).

Starting from a fly line with an X-linked insertion of the above reporter gene, new fly lines with independent reporter gene insertions on an autosome or the X chromosome were created via genetic crosses as described previously [19]. The following mating scheme was carried out with the aim of mobilizing an X-linked transgene, which was linked to the red eye color marker, to a third chromosome containing a *P*-element transposase (*Δ2-3)* gene [28] linked to the stubble bristle (*Sb*) phenotypic marker. The ultimate goal was to then mobilize these new transgene insertions to random locations on the X chromosome and autosomes. Flies containing an X-linked copy of the reporter gene in an otherwise *yellow*, *white* (*yw*) background (yellow body and white eyes) were mated with *yw*; *Δ2-3, Sb/TM6* males. Red-eyed male offspring with stubble bristles then were mated individually to *yw* females. The detection of male offspring with red eyes indicated transgene movement off of the X, since only the female offspring should have inherited an X-linked transgene. If the male offspring had wild type bristles, they were mated individually to *yw* females in order to start new autosomal stocks. If all offspring of these crosses had red eyes and stubble bristles, then the transgene must have moved to the third chromosome containing the transposase gene. In this case, red-eyed males with stubble bristles were mated individually to *yw* females. Offspring that had red eyes and wild-type bristles were mated with *yw* flies of the opposite sex to start a new stock.

To distinguish transgenes located on the X chromosome from those on an autosome, crosses of transgenic males with *yw* females were performed. Males with an X-linked copy of the transgene should transmit the red eye phenotype exclusively to their female offspring, whereas males with an autosomal copy of the transgene should transmit it to 50% of their offspring of both sexes.

### 2.3. Mapping Reporter Gene Insertions

Inverse PCR was performed to determine the exact genomic locations of the transgene insertions [29]. Genomic DNA of each transformed line was extracted from nine flies using the MasterPure DNA Purification Kit (Epicentre, Madison, WI, USA). The genomic DNA was then digested with either HinPI or HpaII, which both cut frequently within the *D. melanogaster* genome. Numerous small fragments were produced and were self-ligated with T4 DNA ligase (New England Biolabs, Ipswich, MA, USA). The fragment containing the inserted transgene was amplified by polymerase chain reaction (PCR) using two primer pairs that matched parts of the of the *pP[wFl]* transformation vector (5′–3′): Plac1-Plac4 (CACCCAAGGCTCTGCTCCCACAAT, ACTGTGCGTTAGGTCCTGTTCATTGTT) and EY.3.F-EY.3.R (CAATAAGTGCGAGTGAAAGG, ACAATCATATCGCTGTCTCAC). The resulting PCR product was sequenced with the primers Sp1 (5′-ACACAACCTTTCCTCTCAACAA-3′) and EY.3.F (above) using BigDye v1.1 chemistry on an ABI 3730 automated sequencer (Applied Biosystems, Foster City, CA, USA). The flanking genomic sequences were then mapped to the *D. melanogaster* reference genome (Release 6.09) with a BLAST search [30] to determine the location of the insertion.

### 2.4. Measuring Reporter Gene Expression

The expression of the *lacZ* reporter gene was measured in heads, testes, and carcasses (defined as the remaining body after the head and testes were removed) with β-galactosidase activity assays. Soluble protein was extracted from five heads, testes or carcasses by homogenizing the tissues/body segments in 200 μL of cold buffer (0.1 M Tris-HCl, 1 mM ethylenediaminetetraacetic acid (EDTA), 7 mM 2-mercaptoethanol; pH 7.5), incubating the homogenate on ice for 15 min, centrifuging at 17,000× *g* for 15 min at 4 °C, and collecting the supernatant, which included the protein extract. Protein extract (50 μL) was combined with 50 μL of 2× assay buffer (200 mM sodium phosphate (pH 7.3), 2 mM MgCl_2_, 100 mM 2-mercaptoethanol, 1.33 mg/mL *o*-nitrophenyl-β-d-galactopyranoside) for each assay. Each biological replicate of a given sample (derived from five flies providing tissue or body segments) was assayed in two technical replicates. For each transformed line and for the corresponding male tissues/body segments from the *yw* strain (used as negative control), 2–3 biological replicates were carried out. β-galactosidase activity was measured spectrophotometrically by tracking absorbance for 50 min at 420 nm at 37 °C. The activity units were defined as the change in absorbance per minute (maximum slope). To test for differences in reporter gene activity between X-linked and autosomal inserts in head, testis and carcass, we performed a Wilcoxon test implemented in R [26] using the mean activity across biological replicates of each transformed line. The mean activity of a biological replicate was calculated as the mean of its two technical replicates.

### 2.5. Maintenance of Drosophila Stocks

All fly stocks were maintained at 22 °C on cornmeal-agar-molasses medium with a 14 h light:10 h dark cycle. All flies used for β-galactosidase assays were 4–6 days old, mated, and either heterozygous (when autosomal) or hemizygous (when X-linked) for the transgene insertion. Thus, all comparisons were of flies carrying exactly two tandem copies of the reporter gene on a single chromosome (i.e., arranged in *cis*).

## 3. Results

### 3.1. Expression of Native Genes on the X Chromosome and Autosomes

To investigate the expression of X-linked and autosomal genes on a genome-wide scale, we used data from FlyAtlas2 [25], which was constructed using RNA-seq data from multiple tissues of each sex. For the testis, we found that the expression of X-linked genes was significantly lower than autosomal genes (Table 1), with the median expression of X-linked genes being 65% that of autosomal genes. This result is consistent with the absence of X-chromosomal dosage compensation in the male germline [15,16]. Although, based on dosage levels alone, one might expect the X chromosome to have 50% of the expression of the autosomes, previous studies have found that, even in the absence of chromosome-wide dosage compensation, the expression of single-dose genes is approximately 65% that of genes present in two doses [15,16,31,32], presumably as the result of gene-specific buffering mechanisms.

In male somatic tissues, there was also a general pattern of X-linked genes having lower expression than autosomal genes, although the reduction in X-chromosomal expression (83–95%) was much less than that seen for the testis (Table 1). This suggests that there is at least nearly complete dosage compensation in male somatic tissues. Indeed, a similar pattern was seen in female somatic tissues, where X-linked expression is 82–98% that of autosomal expression (Table 2), suggesting that the slightly-reduced expression of X-linked genes is not related to dosage compensation. Instead, the reduced expression of X-linked genes in both male and female somatic tissues appears to be the result of the X chromosome being depauperate in genes with very high expression. When highly expressed genes (FPKM > 90; 4–15% of genes depending on tissue) are excluded from the analysis, the ratio of X-to-autosomal expression is much closer to 1 in both sexes (Table 3 and Table 4).

### 3.2. Highly Expressed Genes Are Underrepresented on the X Chromosome

**Figure 1:**
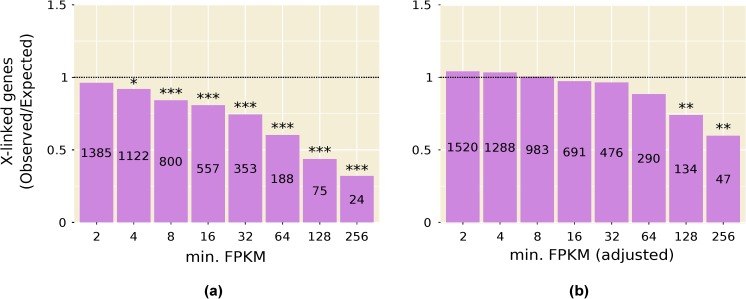
Underrepresentation of highly expressed, X-linked genes in the testis: (**a**) Using the reported expression values for all genes; (**b**) After multiplying the fragments per kilobase of transcript per million mapped reads (FPKM) of all X-linked genes by 1.53 to adjust for the absence of dosage compensation. The number of genes in each expression category (i.e., genes with FPKM greater than or equal to the value on the X-axis) is displayed within the bar. * *p* < 0.05; ** *p* < 0.01; *** *p* < 0.001.

**Figure 2:**
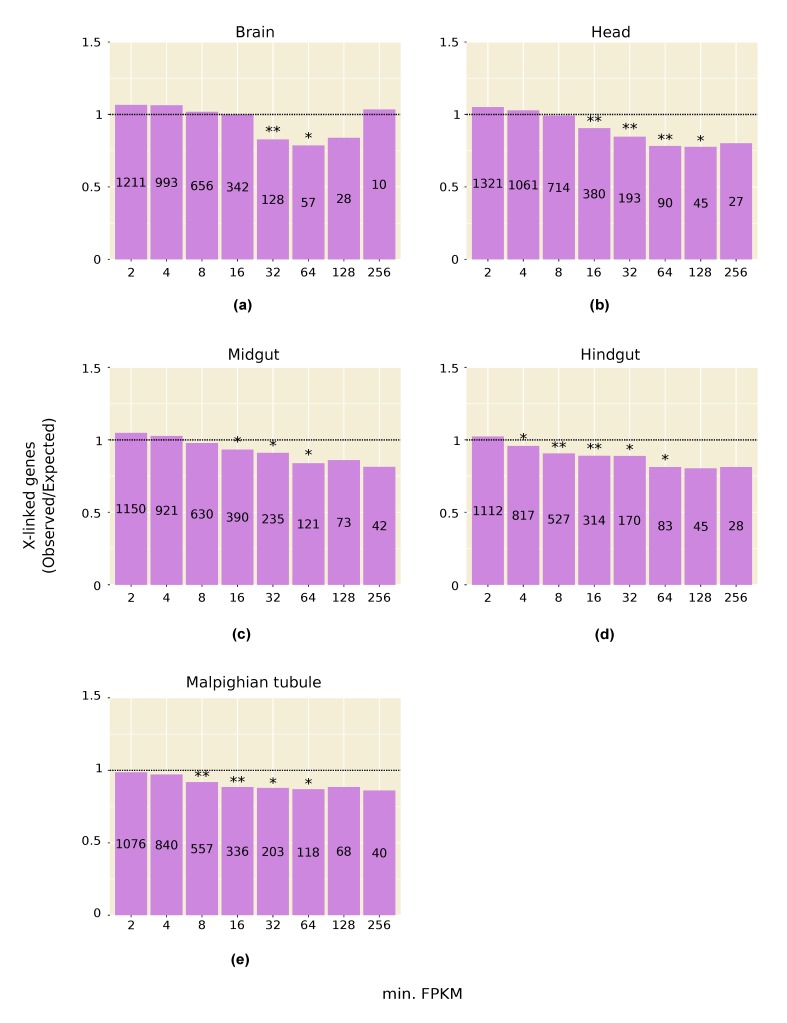
Representation of X-linked genes in male somatic tissues: (**a**) Brain; (**b**) Head; (**c**) Midgut; (**d**) Hindgut; (**e**) Malpighian tubule. The number of genes in each expression category (i.e., genes with FPKM greater than or equal to the value on the X-axis) is displayed within the bar. * *p* < 0.05; ** *p* < 0.01.

Consistent with the above results, we find that genes with high expression in testis, and in other male tissues, are significantly underrepresented on the X chromosome (Figure 1a and Figure 2). This pattern is especially strong in testis and persists even after computationally correcting for the absence of dosage compensation in this tissue: when the expression of all X-linked genes is increased by 1.53-fold to artificially correct for the lack of dosage compensation, there is still a significant underrepresentation of X-linked genes in the highest expression categories (Figure 1b). A correction factor of 1.53 was chosen because it is the inverse of the median X-to-autosome expression ratio (0.65) observed in testis (Table 1). The observed underrepresentation of highly expressed X-linked genes in testis is in line with that reported in previous microarray studies [22,33].

The above patterns appear to be unique to males. In some female somatic tissues, X-linked genes with intermediate expression levels were significantly underrepresented, but not of those within the highest expression classes, while in the ovary there was an enrichment of X-linked genes across most expression classes (Figure S1).

### 3.3. Expression of X-Linked and Autosomal Reporter Genes

**Figure 3:**
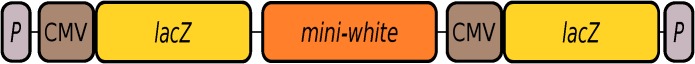
Schematic illustration of the reporter gene construct. The construct contains the *mini-white* marker gene and two copies of the *lacZ* reporter gene under control of the human cytomegalovirus (CMV) promoter. The terminal sequences of the *P*-element (*P*) represent the boundaries of the transgene. The CMV promoter is able to drive ubiquitous expression of the reporter gene in *D. melanogaster*.

In order to compare the X-linked and autosomal expression of the same gene in multiple tissues, we generated transgenic flies carrying a ubiquitously-expressed reporter gene (Figure 3). We obtained 29 transgenic lines, each with the reporter gene inserted at a unique genomic location. Twelve of the lines had autosomal insertions, which were located on all possible chromosomal arms, including the fourth chromosome (Table S1). Seventeen of the lines had X-linked insertions. These were distributed widely along the X chromosome, except for two inserts that were separated by a distance of only 320 bp (Table S1).

**Figure 4:**
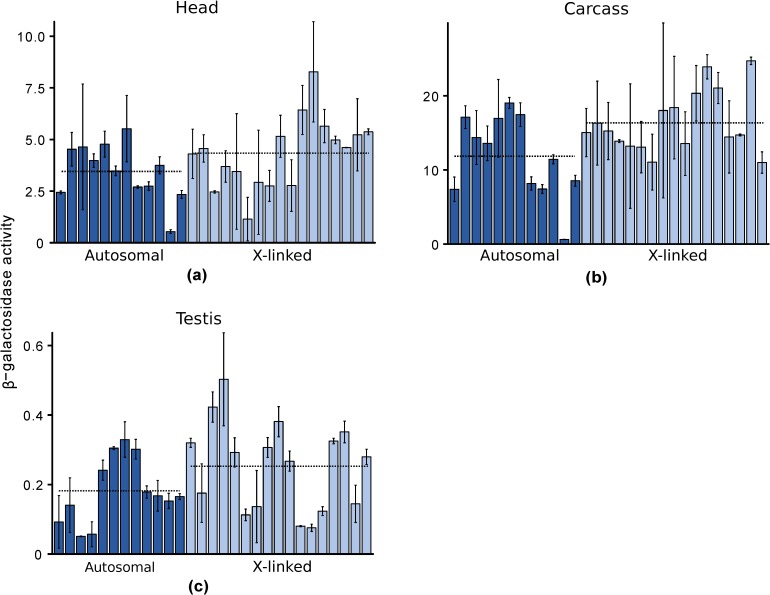
Autosomal and X-linked expression of a ubiquitously-expressed reporter gene in males: (**a**) Head; (**b**) Carcass; (**c**) Testis. Each bar represents a transformed line with the reporter gene inserted at a unique autosomal (dark blue) or X-linked (light blue) location. Expression was measured spectrophotometrically as β-galactosidase activity in units of mOD/min. Error bars indicate the standard deviation across biological replicates. Dotted lines indicate the average activities of all autosomal or X-linked lines.

In male heads, the mean (median) β-galactosidase activities of autosomal and X-linked lines were 3.45 (3.61) and 4.34 (4.56) mOD/min, respectively, and did not differ significantly (Wilcoxon test, *p* = 0.14) (Figure 4a). Even after removing an outlier line with an autosomal insert (A39 in Table S1) that had much lower expression (0.54 mOD/min) than the other lines, the difference between autosomal and X-linked expression remained non-significant (Wilcoxon test, *p* = 0.24), although expression of the X-linked reporter genes was slightly higher than that of the autosomal reporter genes. Hence, there was no evidence for X suppression in male heads. In male carcasses, the mean (median) β-galactosidase activities of autosomal and X-linked lines were 11.85 (12.52) and 16.36 (15.04) mOD/min, respectively, and did not differ significantly (Wilcoxon test, *p* = 0.06) (Figure 4b). Even excluding the outlier autosomal line (A39, with expression of only 0.62 mOD/min), there was no significant difference between autosomal and X-linked expression (Wilcoxon test, *p* = 0.11). Hence, there was no evidence for X suppression in male carcasses. Again, the expression of the X-linked reporter genes was slightly higher than that of the autosomal ones.

In testis, the mean (median) β-galactosidase activities of autosomal and X-linked lines were 0.18 (0.17) and 0.25 (0.28) mOD/min, respectively, and did not differ significantly (Wilcoxon test, *p* = 0.2) (Figure 4c). Hence, there was no evidence for X suppression in the male germline. Similar to heads and carcasses, we observed slightly higher expression of the X-linked reporter genes in testis. Although reporter gene expression was relatively low in testis, it was well above that of a non-transgenic line, which had a mean (median) β-galactosidase activity of −0.05 (−0.05) with a standard deviation of 0.07. Thus, we can conclude that the reporter gene is being expressed in the testis, albeit at a low level that does not differ between the X chromosome and the autosomes.

## 4. Discussion

Transcriptomic studies of *D*. *melanogaster* have found that, on average, X-linked genes are expressed at lower levels than autosomal in testis [15,16] and that there is a deficit of highly expressed X-linked genes in this tissue [22,33]. These results are confirmed by our analysis of a new tissue-specific expression dataset (Table 1, Figure 1). In addition, we find that the above patterns are present in multiple male somatic tissues, albeit to a weaker extent than in testis (Table 1, Figure 2). A similar reduction in X-linked expression has been reported in mammals, where it has been attributed to transcriptional traffic jams caused by the hemizygosity of the X chromosome in males [34].

Although previous reporter gene studies have suggested that the expression of the X chromosome is greatly suppressed (typically 3–7-fold) in the male germline [19,21,35], the expression of endogenous X-linked genes in testis is not much lower than that of autosomal genes. Our analysis of native genes indicates that the median X-to-autosomal expression in testis is about 0.65 (Table 1), which is similar to previous reports [15,16] and consistent with the expectation for a lack of X chromosome dosage compensation in this tissue. Below we discuss two factors that likely mitigate the effects of X suppression on native genes and might explain why a greater signal of X suppression is not observed in genome-wide expression studies.

**Figure 5:**
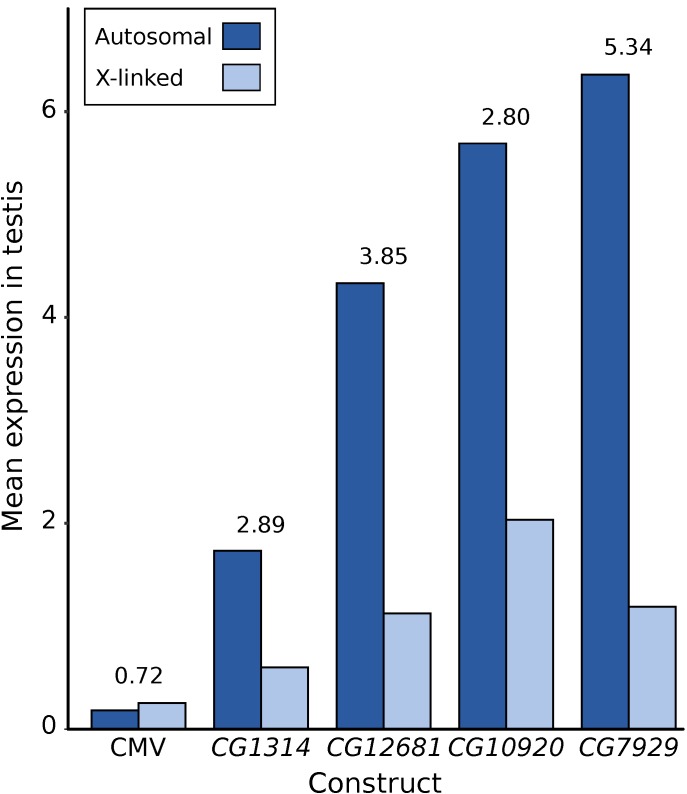
Degree of X suppression in the male germline of *D. melanogaster* as revealed by reporter gene constructs. The mean X-linked (light blue) and autosomal (dark blue) reporter gene expression, as well as the ratio of autosomal-to-X expression (number above the bars), is shown. Expression data for the CMV construct are from the current study, while those for the other four testis-specific constructs were published previously [21,35]. The expression of three constructs (*CG1314*, *CG12681*, *CG10920*) was normalized by a factor of 5/6, as six flies were used per assay in contrast to five flies for the other constructs.

**Figure 6:**
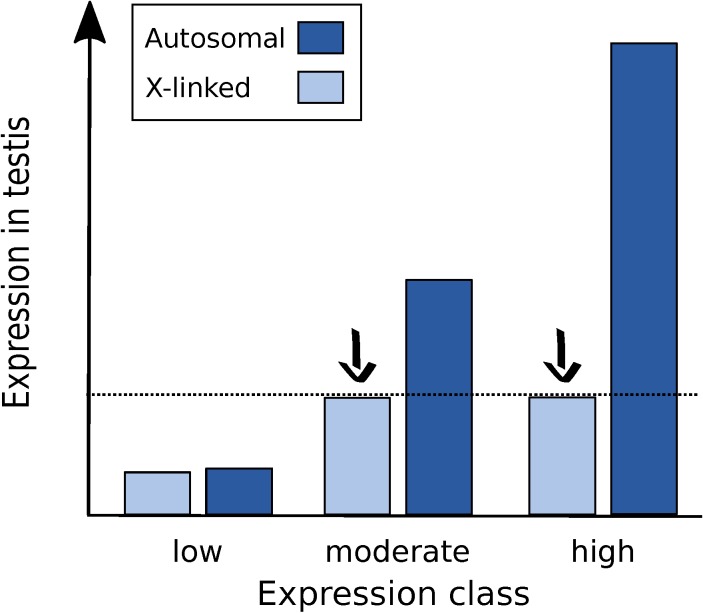
Proposed model for the regulation of the X chromosome in the male germline of *D. melanogaster*. X suppression (black arrows) affects genes that are expressed above a certain threshold (dashed line). Genes that have the potential for high expression on the autosomes, such as housekeeping and testis-specific genes, are suppressed when X-linked. The degree of X suppression is dependent on a gene’s autosomal expression class, with the highly expressed genes being more severely affected. Genes that have low expression are not affected by X suppression, as they do not reach the expression threshold.

First, X suppression appears to be strongest for highly expressed genes and, thus, may affect only a minority of the genes in the genome. For native genes located within regions of the X chromosome that were transposed to autosomes, Landeen and colleagues found a significantly positive correlation between expression level in testis and the degree of X suppression [22]. Similarly, for the reporter genes tested to date, there is a strong correlation between a gene’s expression level in testis when it is autosomal and the degree of X suppression that is observed (Figure 5). Even with a limited sample size of five reporter genes, the correlation between autosomal expression and the degree of X suppression is strong and significant (*R²* = 0.75, *p* = 0.036). This relationship may explain why the X chromosome shows a significant underrepresentation of highly expressed genes in the testis, even after accounting for the reduction in expression attributable to the lack of dosage compensation (Figure 1b) and suggests that there may be a minimal threshold expression level that must be met before suppression occurs (Figure 6). This could explain why the *CMV-lacZ* reporter gene, which has low expression in testis, does not show reduced expression when it is located on the X chromosome (Figure 4c and Figure 5).

Another important factor that mitigates the suppression of native X-linked genes in the male germline is the accumulation of regulatory motifs that enhance their expression in the testis. Landeen and colleagues identified a 7-bp regulatory motif that is enriched in the upstream region of X-linked, testis-specific genes and experimentally verified it to be a testis enhancer [22]. Interestingly, this motif is present in two copies in the immediate upstream region of *CG12681* and is included in one of the reporter genes showing significant X suppression (Figure 5). Because the regulatory motif functions equally well on the X chromosome and autosomes [22], movement of the *CG12681* reporter gene from the X chromosome to an autosome does not diminish its efficacy as a positive regulator of testis expression [21].

Species with heteromorphic sex chromosomes often evolve mechanisms to inhibit the expression of the X (or Z) chromosome in the gonads of the heterogametic sex. For instance, MSCI is present in several species, including mammals with XY males, chicken with ZW females [36], and grasshopper [37] and *Caenorhabditis elegans* with X0 males [38]. X suppression in *Drosophila* does not appear to be completely analogous to MSCI, because MSCI is marked by a massive switch-off of most X-linked genes [39], while X suppression strongly inhibits only the genes with high expression in testis. It is also possible that the two mechanisms act at different developmental stages of spermatogenesis [15,40].

Why is it necessary to evolve a mechanism to inhibit expression of the sex chromosomes in the heterogametic sex? Hypotheses proposed as evolutionary explanations for this process include the avoidance of (i) non-homologous recombination; (ii) sexual antagonism; and (iii) meiotic drive. Under the first hypothesis, recombination events are regularly suppressed between the non-homologous sex chromosomes, as predicted by the Haldane-Huxley rule [41], to prevent the potentially deleterious outcomes of aneuploidy, such as developmental and mental retardation in humans [42]. Under hypothesis (ii), since the X chromosome spends two thirds of its evolutionary time in females, it could favour the evolution of genes whose expression optimum is beneficial in females, but deleterious to males, leading as a consequence to an inhibition mechanism evolving to suppress the expression of genes that are detrimental to male fitness [43]. The third hypothesis proposes that inhibition of the sex chromosomes could have evolved as a defense mechanism against alleles of a selfish gene that can induce destruction of the portion of gametes that do not carry it. Numerous cases of sex-chromosome meiotic drive have been documented in *Drosophila* species [44,45,46] and in other insects, such as stalk-eyed flies [47], where the presence of a driving X chromosome in males leads to eradication of the gametes bearing the Y chromosome [48]. 

Our results indicate that the regulation of X-chromosomal gene expression differs between germline and somatic tissues. In the male germline, expression of the X chromosome is generally lower than that of the autosomes due to the absence of dosage compensation and the suppression of X-linked genes that otherwise would be highly expressed. A limitation of the current study is that the expression of native and reporter genes was measured in whole testes. Thus, expression differences between cell types or developmental stages within this tissue could not be detected [23]. Further studies of single-cell expression or fine-scale patterns of reporter gene expression may shed light on this issue. Additionally, the molecular mechanism responsible for suppression of the X chromosome in the male germline remains to be elucidated. Future research could therefore concentrate on the identification of genes involved in this process through genetic screens or candidate gene analyses. Another area for future research is determining how the specific sequence context of a gene’s location on the X chromosome, such as its distance to a binding site of the DCC, affects its expression in germline and somatic tissues [49]. This topic could be investigated by comparing the expression of a large number of reporter gene insertions spanning the X chromosome at high density in multiple tissues. 

In conclusion, the unique selection pressures acting on sex chromosomes, in particular the X chromosome, have resulted in the evolution of new chromosome-specific gene regulatory mechanisms. This regulation varies among tissues and between sexes and contributes to global differences in gene content and expression between the X chromosome and the autosomes.

## Figures and Tables

**Table 1:** Expression of all genes in male tissues.

Tissue	X Genes	X Median	A Genes	A Median	X/A	*p*
Testis	1581	8.16	8657	12.46	0.65	<2.2−16
Brain	1355	7.73	6871	8.16	0.95	0.22
Head	1471	7.64	7595	8.35	0.91	0.007
Midgut	1268	7.88	6649	8.94	0.88	0.10
Hindgut	1249	6.27	6661	7.58	0.83	0.0006
Tubule	1258	6.76	6962	7.43	0.91	0.08

X = X chromosome; A = autosome; median = median fragments per kilobase of transcript per million mapped reads (FPKM); X/A = ratio of median FPKM values; *p* = *p*-value of Wilcoxon test comparing X versus A expression.

**Table 2:** Expression of all genes in female tissues.

Tissue	X Genes	X Median	A Genes	A Median	X/A	*p*
Ovaries	1206	10.78	5723	10.52	1.02	0.56
Brain	1356	7.82	6904	7.98	0.98	0.90
Head	1474	7.99	7580	8.50	0.94	0.05
Midgut	1194	6.82	6065	8.31	0.82	0.004
Hindgut	1289	7.74	6687	8.91	0.87	0.01
Tubule	1226	7.82	6332	8.84	0.88	0.12

**Table 3:** Expression of all genes, excluding those with FPKM > 90, in male tissues.

Tissue	X Genes	X Median	A Genes	A Median	X/A	*p*
Testis	1460	7.17	7245	8.99	0.80	2.4−05
Brain	1317	7.47	6594	7.72	0.97	0.59
Head	1408	7.21	7127	7.60	0.95	0.09
Midgut	1171	7.10	6026	7.44	0.95	0.47
Hindgut	1188	5.89	6233	6.87	0.86	0.05
Tubule	1164	5.97	6391	6.44	0.93	0.13

**Table 4:** Expression of all genes, excluding those with FPKM > 90, in female tissues.

Tissue	X Genes	X Median	A Genes	A Median	X/A Median	*p*
Ovaries	1158	10.34	5485	10.09	1.02	0.47
Brain	1313	7.54	6638	7.57	1.00	0.78
Head	1398	7.44	7113	7.74	0.96	0.15
Midgut	1108	6.35	5521	7.14	0.89	0.03
Hindgut	1213	7.20	6196	7.90	0.91	0.06
Tubule	1121	6.88	5750	7.44	0.92	0.17

## Supplementary Materials

The following are available online at http://www.mdpi.com/2073-4425/9/5/242/s1, Figure S1: Representation of X-linked genes in female tissues, Table S1: Genomic locations of the transgene insertions.
