# Supplementary material for: Regulation of the X Chromosome in the Germline and Soma of Drosophila melanogaster Males
Source: Genes (Basel). 2018 May 4;9(5):242. doi: 10.3390/genes9050242 (PMC5977182; doi:10.3390/genes9050242)
Supplement: Supplementary file 1 [file genes-09-00242-s001.zip › Figure S1.pdf]

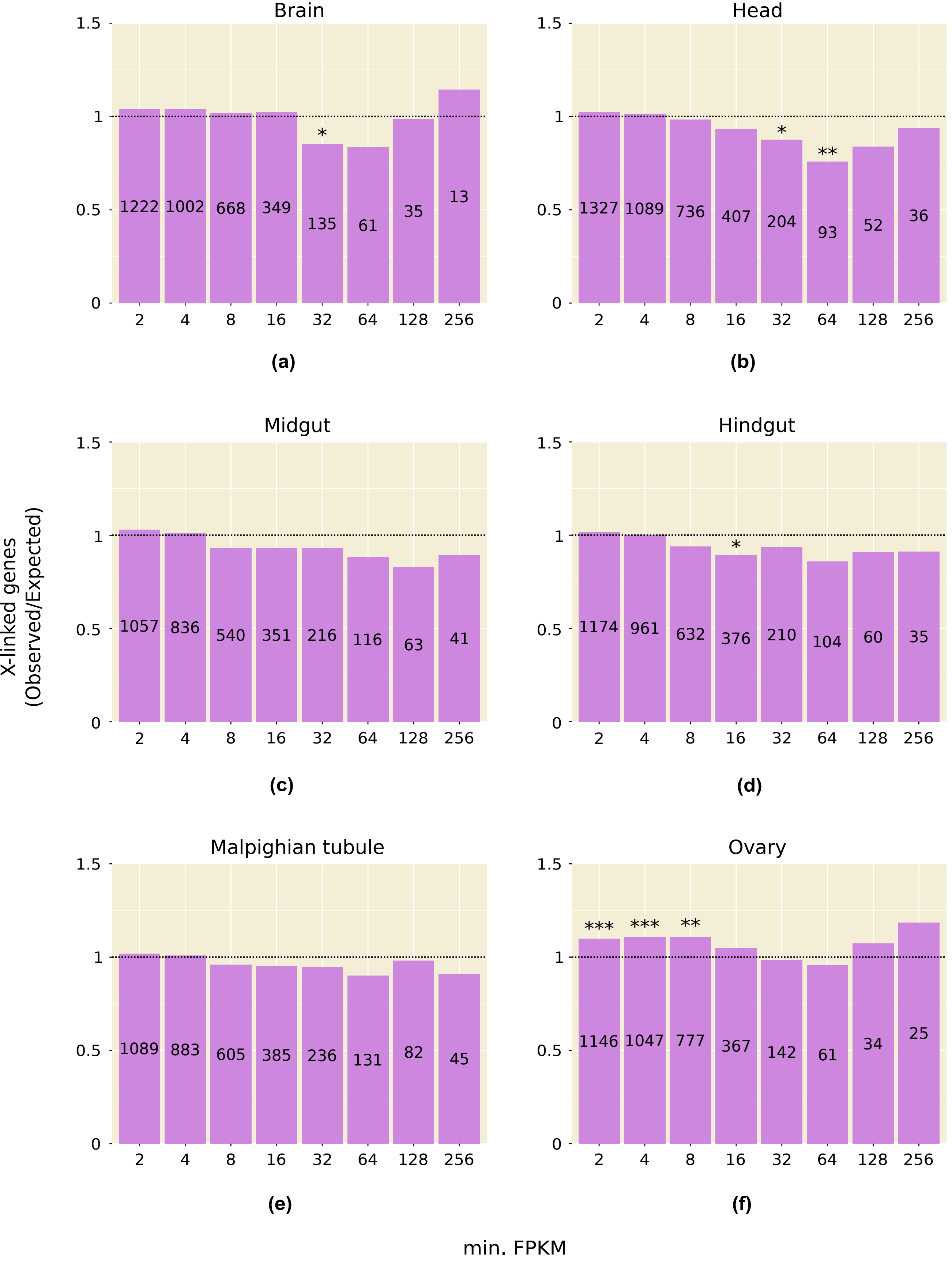

Figure S1. Representation of X-linked genes in female tissues: (**a**) Brain; (**b**) Head; (**c**) Midgut; (**d**) Hindgut; (**e**) Malpighian tubule. The number of genes in each expression category (i.e., genes with FPKM greater than or equal to the value on the X-axis) is displayed within the bar. * $p < 0.05$; ** $p < 0.01$; *** $p < 0.001$.
