# Supplementary material for: Regulation of the X Chromosome in the Germline and Soma of Drosophila melanogaster Males
Source: Genes (Basel). 2018 May 4;9(5):242. doi: 10.3390/genes9050242 (PMC5977182; doi:10.3390/genes9050242)
Supplement: Supplementary file 1 [file genes-09-00242-s001.zip › Table S1.pdf]

Supplementary Table S1. Genomic locations of the transgene insertions.

Line ID	Chr	Cyt. band	Coordinate	Location	Affected gene	Proximal gene	Distal gene
A6	3R	88D5	14817989	intron/exon	btsz		
A10	3L	68E1	11821852	exon/intron, exon	CG5946, CG11597	CG14130, CG42255	Rpl10Ab, CG32095, CycA, CG7264
A13	3R	90C5	17794503	5'UTR	CG43102		pasi1, CG7379, CG17803
A14	3R	93F14	21856246	5'UTR	glec	Gr93d	lsn
A16	2R	55D1	18504287	inter		CG30116	GstE11
A26	2L	30B10	9521294	inter		CG33298	Oatp30B, CG31883
A27	2R	44F6	8913824	intron	Pgi	CG8252, CG30349, CG8258	lin, CG34219, CG8248, Spt
A28	2L	31A2	10057376	5'UTR	Pen	Cpr31A, CG33301	Spn31A, CG44153
A29	2L	35D4	15762783	inter		Gli, l(2)35Df	CG3793, wek, Ku80, CG31826
A33	3L	70A3	13227763	inter			caps
A39	4	102D4	704530	intron	ey	myo	
A40	3L	61C8	699829	inter		CG32483, RabX6, Vti1a, CG13894	CG13895, CkIIalpha-i3
2X	X	6E4	6998549	inter		Inx7, ogre	Inx2
X1	X	9B6	10318800	intron	alpha-Man-la		CG2909, Gip
X5	X	12F4	14826069	5'UTR	rut		CG14408, CG14411
X8	X	2B13	1873539	exon	Pgam5	CG14803, Pex5, MED18, CG14814	Vsp26, CG14817, CG14805, CG14818, CG14806, trr
X9	X	13A9	15075124	intron	Lsd-2	dob, opm, ND-B18	CG33177, CG33178, CG9065

X11	X	8B6	8894224	intron	Moe		CG1885, Rbm13, e(r)
X15	X	3E6	3720051	inter		Rala	Tlk
X20	X	3D3	3451351	inter		CG12535	
X21	X	6E4	6968661	3'UTR, 5' UTR	CG14431, CG32732		CG4586
X22	X	12C1	13762705	inter		Yp3, Rtc1, CG32625	rdgB
X23	X	10C5	11558520	5'UTR/intron	CG1572	Drak	PGRP-SA, RpII215
X24	X	19F4	21319242	5'UTR	SLIRP1	CG33713, Rpt6, CG1801	Dd, CG1486
X25	X	12E5	14222240	intron	l(1)G0007		CG11674, mRpL38
X26	X	7D5	8057219	intron	fs(1)h		mys
X27	X	6E4	6998869	5'UTR/intron	Inx2	Inx7	
X28	X	18F4	19887117	3'UTR	amn	Hers	Hers
X29	X	1E4	1209204	inter		CG14625, CG11381, CG14624	CG11382, CG11398, CG3638

Chr = Chromosomal location; Cyt. band = Cytological band; Coordinate = Coordinate based on *D. melanogaster* reference genome Release 6.09; Location = Type of genomic location, ”inter” indicates intergenic regions; Affected gene = Gene overlapping with the transgene insertion; Proximal/Distal gene = Gene found within 10kb of the transgene insertion
